# Can ultrasonographic measurement of respiratory variability in the diameter of the internal jugular vein and the subclavian vein predict fluid responsiveness in parturients during cesarean delivery? A prospective cohort study

**DOI:** 10.1016/j.heliyon.2022.e12184

**Published:** 2022-12-13

**Authors:** Shaobing Dai, Jianjun Shen, Xia Tao, Xinzhong Chen, Lili Xu

**Affiliations:** a Department of Anaesthesiology, Women’s Hospital, Zhejiang University School of Medicine, Hangzhou, Zhejiang Province, China; b Department of Anaesthesiology, The Second Affiliated Hospital, School of Medicine, Zhejiang University, Hangzhou, Zhejiang Province, China; c Department of Ultrasound, Women’s Hospital, Zhejiang University School of Medicine, Hangzhou, Zhejiang Province, China

**Keywords:** Internal jugular vein, Subclavian vein carotid artery, Doppler ultrasound, Fluid therapy, Respiration

## Abstract

**Background:**

Ultrasonic measurements of the respiratory variability in the diameter of internal jugular vein (IJV-CI) and subclavian vein (SCV-CI) have recently been reported to be useful in predicting fluid responses in non-obstetric patients with spontaneous respiration. This study evaluated whether IJV-CI and SCV-CI could reliably predict fluid responsiveness in parturients undergoing elective cesarean delivery.

**Objective:**

This study was conducted to determine whether two indicators measured by ultrasound are good predictors of fluid responsiveness of parturients with spontaneous respiration in elective cesarean delivery.

**Design:**

A prospective cohort study.

**Setting:**

A single-center tertiary specialty hospital in China.

**Patients:**

A total of 86 patients scheduled for elective cesarean section were included and 6 were excluded for various reasons.

**Interventions:**

Based on the results of the fluid challenge, the included parturients were divided into two groups, with those who responded to fluid challenge defined as the positive group and those who did not respond defined as the negative group.

**Main outcome measures:**

The primary endpoint was to determine the predictive value of IJV-CI and SCV-CI for fluid responsiveness (≥15% increases in SVI after fluid challenge) in spontaneous respiration patients.

**Results:**

Forty-three (53.8%) parturients were fluid responders. IJV-CI and SCV-CI proved to be the independent predictors for fluid responsiveness by multivariate logistic regression. The area under the ROC curve for IJV-CI and SCV-CI were 0.881 (95% CI, 0.808–0.953, p < 0.0005) and 0.757 (95% CI, 0.648–0.865, p < 0.0008), respectively. Their optimal cut-off values for IJV-CI and SCV-CI were 20.7% (sensitivity of 60%; specificity of 79%) and 32.0% (sensitivity of 34%; specificity of 96%), respectively. The grey zone for IJV-CI and SCV-CI for fluid responsiveness were 20.4–32.4% and 30.4–44.6% and included 25% and 23% of the patients, respectively.

**Conclusion:**

Ultrasonic-derived IJV-CI is better than SCV-CI in predicting fluid responsiveness in spontaneously breathing parturients. Both IJV-CI and SCV-CI are the accurate and readily accessible indices of fluid responsiveness in parturients undergoing elective cesarean delivery.

**Trial registration:**

chictr.org.cn (ChiCTR1900028450).

## 1 Introduction

Nowadays, some static and dynamic parameters mainly reflecting the cardiopulmonary interaction have been widely used to predict fluid responsiveness and to assess response to intravenous infusion resuscitation. Importantly, echocardiography has been shown to assess fluid responsiveness by viewing the internal structure and the blood flow of the heart and great vessels and to guide fluid resuscitation in critically ill patients [1, 2]. Unfortunately, both the compression of the inferior vena cava by the pregnant uterus and the technical difficulty in identifying by ultrasound limit the widespread use of the respiratory variability of deep abdominal and thoracic veins, such as superior vena cava (SVC) to predict fluid reactivity during cesarean section [3, 4]. Interestingly, present evidence has shown that when compared with respiratory variability of inferior vena cava diameter (IVC-CI), respiratory variability of internal jugular vein variability (IJV-CI) and subclavian vein (SCV-CI) appear to be the suitable metrics of fluid responsiveness in spontaneously breathing patients with sepsis and acute circulatory failure [1, 5, 6].

As we know, physiological and hemodynamic changes during pregnancy may uncover previously unrecognized heart and great vessels disease and lead to significant morbidity and mortality [7, 8]. Moreover, spinal anesthesia for cesarean section induces sympathetic block, decreased systemic vascular resistance (SVR), and increased venous volume, leading to relative hypovolemia. Therefore, the assessment of maternal volume status and fluid management play a vital role in the perioperative management of obstetric anesthesia [9]. However, it is still unclear whether ultrasonic measurements of IJV-CI and SCV-CI could be the reasonable variables of intravascular volume status and play a key guiding role in perioperative fluid therapy in parturients with spontaneous breathing. Our study aimed to investigate their predicting ability for fluid responsiveness in parturients undergoing elective cesarean delivery.

## 2 Methods

### 2.1 Patients

Our prospective cohort study was ratified by the Research Ethics Committee in Women’s Hospital, Zhejiang University School of Medicine (IRB no. 20191119) on 19 November, 2019 and then signed up at the Chinese Clinical Trial Registry (ChiCTR) (www.chictr.org) (ChiCTR1900028450) on 2020/12/01. Subsequently, we carried out it at the Women’s Hospital, School of medicine, Zhejiang University from 1 January, 2020 to 31 March, 2020.

After written informed consent was obtained, 80 American Society of Anaesthesiologists (ASA) class I-II elective cesarean sections aged 18 years or older were enrolled. Parturients with arrhythmias, preeclampsia, gestational hypertension, internal jugular or subclavian venous thrombosis, severe mitral or tricuspid regurgitation, severe aortic regurgitation, cardiac dysfunction, cerebrovascular disease, pulmonary edema, impaired cervicothoracic echo, or diabetes were excluded from our study.

### 2.2 Anesthesia management

In the operating room, patients are monitored using standard monitoring including the electrocardiogram (ECG), pulse oxygen saturation (SpO2), heart rate (HR), and mean arterial pressure (MAP). Each patient received two-point combined spinal and epidural Anesthesia in the left lateral decubitus position. Specifically, epidural Anesthesia was carried out by puncture catheterization in the lumbar intervertebral space 1–2 with 2% lidocaine 5 ml, subarachnoid Anesthesia was performed in lumbar intervertebral space 3–4 puncture with ropivacaine 15 mg specific gravity solution. Postoperatively, each patient received a patient-controlled epidural analgesia pump with 100 ml 0.2% ropivacaine.

### 2.3 Study protocol

In each patient, IJV-CI, SCV-CI, and hemodynamic parameters were measured immediately after fetus delivery and 10 min after a fluid loading of 6 ml/kg of 6% hydroxyethyl starch 130/0.4. Ultrasound examinations were taken using a SONIMAGE HS1 ultrasound device (Konica Minolta Inc, Shanghai, China), equipped with a 6–13 MHz variable frequency linear transducer (L12-3 Broadband Linear Array Transducer). IJV-CI of internal jugular vein was measured by two independent sonographers who were blinded to each other’s Doppler results and hemodynamic variables of the patients. The optimal short-axis view of IJV was visualized by placing the ultrasound transducer perpendicular to the skin in the transverse plane on the neck at the level of the cricoid cartilage on the B-mode real-time image (Figure 3). After the ultrasound probe was rotated 90° and the optimal view of the longitudinal axis was gained. The IJV diameter at the end of expiration (IJVexp) and inspiration (IJVins) respectively over a whole respiratory cycle were measured and IJV-CI (%) = (IJVexp-IJVins)/IJVexp × 100 [10]. The average values from three consecutive respiratory periods were adopted for analysis. The optimal short-axis view of SCV was visualized by placing the ultrasound transducer perpendicular to the clavicle long axis so that a short portion of the vein and the subclavian artery could be visualized in the same view. Then, the probe was rotated parallel to the clavicle to gain a longitudinal view of the SCV. The SCV diameter at the end of expiration (SCVexp) and inspiration (SCVins) respectively over a whole respiratory cycle were measured and SCV-CI(%) = (SCVexp-SCVins)/SCVexp × 100 [6].

Echographic measurements of the diameter of the left ventricular outflow tract were obtained while a 1.5–4.5 MHz phased array probe was placed at the left margin of the sternum between the two and three costal and determined by using the images of the largest opening of the aortic valve. All values represented the mean of three consecutive measurements and the mean of the two sonographers was employed for analysis. Sonographic assessment of the aortic flow time velocity integral was gained when the probe was put at the apex and determined by the mean of five consecutive beats of a complete respiratory cycle using the images of the apical five-chamber of the aortic annulus. SVI, the left ventricular outflow tract area, and BSA were calculated as the formulas: SVI = (left ventricular outflow tract area × aortic flow time velocity integral)/body surface area (BSA), the left ventricular outflow tract area = π × (left ventricular outflow tract diameter/2) [11], BSA (m^2^) = 0.0061 × body length (cm) + 0.0128 × body weight (kg) − 0.1529 [12].

### 2.4 Study endpoint

The value of IJV-CI and SCV-CI for predicting fluid responsiveness (≥15% increases in SVI after fluid challenge) in spontaneous respiration patients [13] was the primary endpoint.

### 2.5 Statistical analysis

The area under the receiver operating characteristic (AUROC) curve of IJV-CI for predicting fluid responsiveness was 0.88 as in the previous study [14], we assumed the AUROC curve of IJV-CI was 0.75. We required at least 42 patients to detect a difference of 0.25 between the AUROC curves of IJV-CI (0.75) and SCV-CI (0.5), with a type I error of 0.05 and an 0.9 power [14]. A sample size of 46 patients was needed as a possible 10% dropout rate. Volume responsiveness was defined as an increase of 15% or more in SVI after fluid challenge. The normality of the data distribution was determined by Shapiroe-Wilk and Kolmogorove-Smirnov tests. If data were normally distributed or not, continuous variables were expressed as mean (standard deviation) or median (interquartile range) while categorical variables were expressed as absolute numbers (%). For comparisons between responder and non-responder, a paired t-test was used for normally distributed data, Manne Whiney U-test was applied for non-normally distributed data, and the X^2^ test or Fisher’s exact test was utilized for categorical variables. Multivariate logistic regression analyses were used to evaluate multivariate predictors of fluid responsiveness. The ability of IJV-CI and SCV-CI in predicting liquid reaction was evaluated using the receiver operating characteristic (ROC) curve. Maximized Youden’s index (J = Sensitivity + Specificity − 1 = Sensitivity-False-Positive Rate) was used to determine the “optimal” cut-off values were tested by using [15] a correlation value of 90% sensitivity and 90 specificity was applied to assess the cut-off values defining the gray area [16]. Moreover, the intra-observer variability (repeatability) and inter-observer variability (reproducibility) were evaluated for all patients assessed for IJV-CI and SCV-CI and variability was determined by dividing the absolute difference between the two values by their mean. Accordingly, the inter-observer repeatability of IJV-CI and SCV-CI in all data sets can also be identified by calculating the coefficient of variation (CV) and intraclass correlation coefficient (ICC). The Bland-Altman plot was used to assess the inter-observer agreement of IJV-CI and SCV-CI. SPSS 23 (SPSS Inc., Chicago, IL, USA) was used for statistical analysis and PASS 14.0.5 (NCSS Statistical Software, Kaysville, UT, USA) was applied for calculating the sample size. The 95% confidence interval (CI) was figured out and P-value < 0.05 (two-tailed) was considered statistically significant.

## 3 Results

### 3.1 Patients

**Figure 1:**
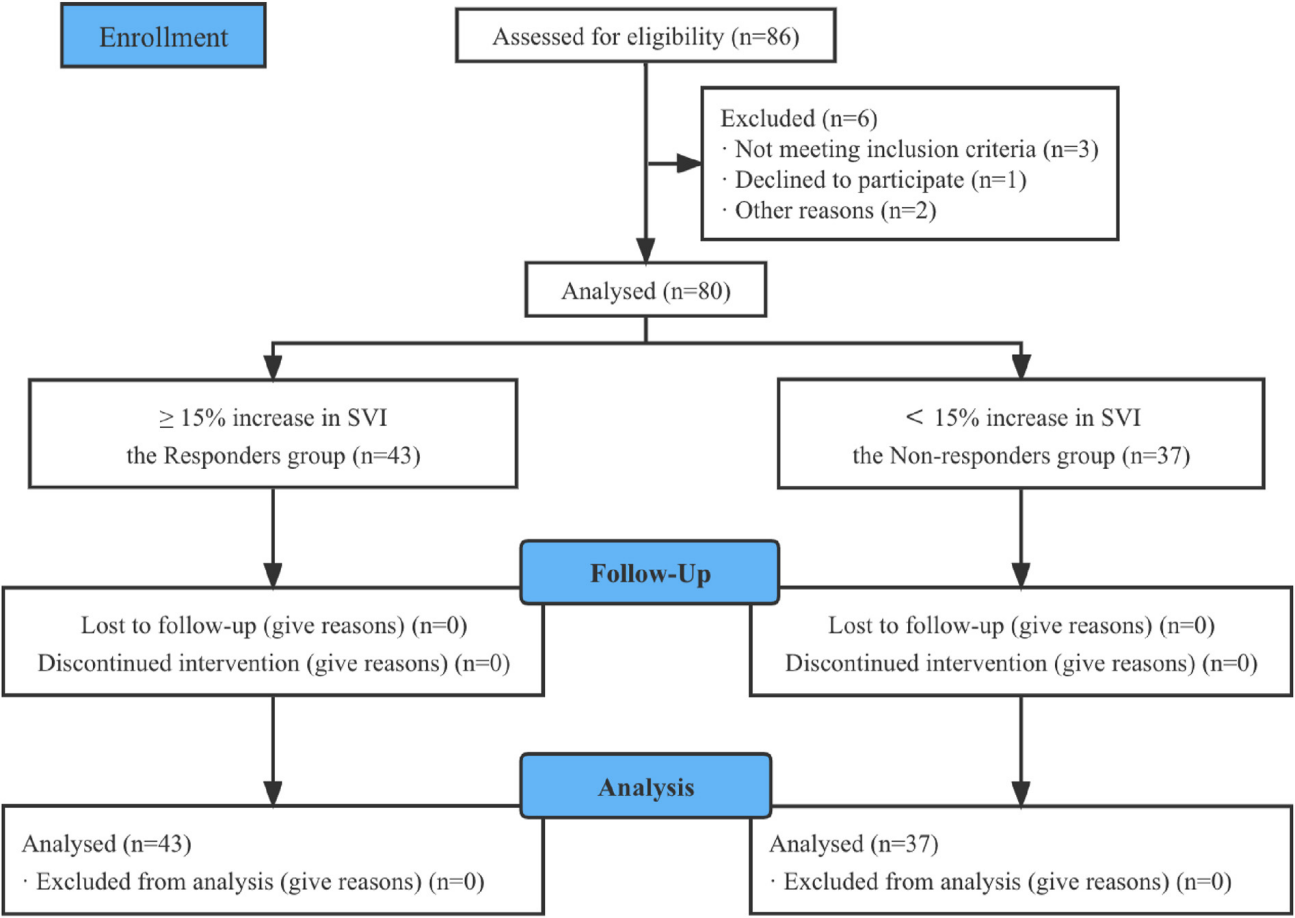
Subject selection process. A total of 86 patients fit inclusion criteria, 6 patients excluded, 80 patients approached for consent, 80 patients studied, 43 patients in the Responders group, 37 patients in the Non-responders group.

Of the 86 eligible patients recruited, 6 were excluded because they did not meet the inclusion criteria (n = 3), refused to participate (n = 1), and for other reasons (n = 2). Therefore, 80 subjects were enrolled for the final analysis (Figure 1). There was no significant difference between the responders group (n = 43) and the non-responders group (n = 37) (Table 1).

**Table 1:** Patient characteristics.

	Responders group (n = 43)	Non-responders group (n = 37)	P value
Age (yr)	32.0 ± 4.5	31.7 ± 3.8	0.732
Height (cm)	160.3 ± 5.9	159.6 ± 5.4	0.581
Weight (kg)	70.2 ± 7.6	68.3 ± 6.7	0.255
BMI	27.3 ± 3.0	26.9 ± 2.7	0.448
Duration of Surgery (min)	54.0 ± 17.5	54.2 ± 13.5	0.945
Gestational age (weeks)	38.5 ± 1.1	38.2 ± 2.1	0.087
Parity	1.4 ± 0.7	1.3 ± 0.9	0.511

Values are numbers or means ± SD.

BMI: Body mass index (kg/m^2^).

∗p < 0.05 compared with Responders group.

### 3.2 Hemodynamic variables before and after fluid challenge

**Figure 2:**
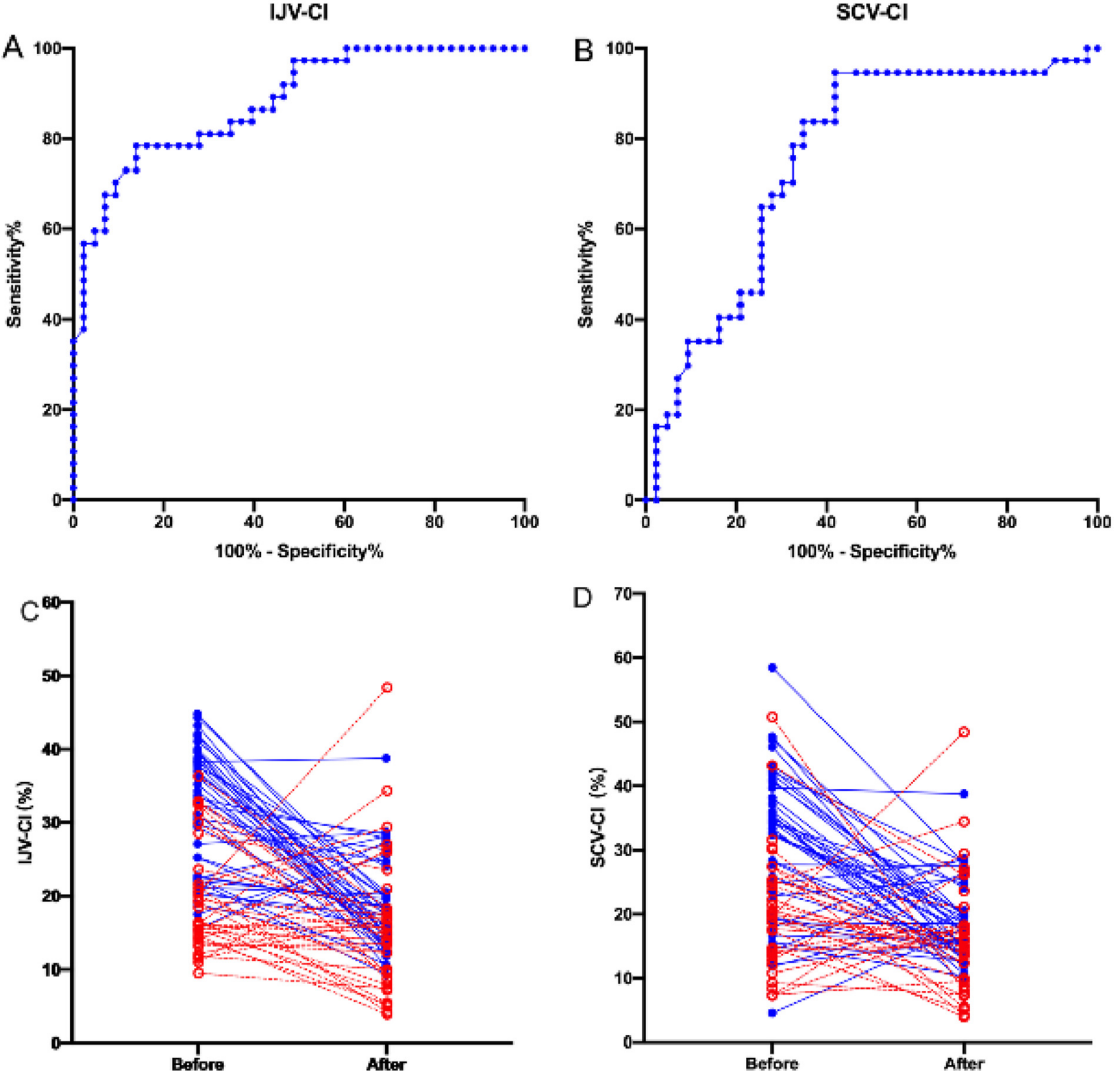
Individual responses to fluid challenge and ROC curve for IJV-CI and SCV-CI. Upper row: individual responses to fluid challenge for IJV-CI (A) and SCV-CI (B). Responders are presented as blue full line and closed circles; Non-responders are presented as red dashed line and open circles. Lower row: receiver operating characteristic curves showing the ability of IJV-CI (C), and SCV-CI (D). The area under the ROC curve for IJV-CI and SCV-CI were 0.881 (95% CI, 0.808–0.953) and 0.757 (95% CI, 0.648–0.865), respectively. AUROC, area under the receiver operating characteristic; CI, confidence interval; IJV-CI: the respiratory variability in the diameter of internal jugular vein; SCV-CI: the respiratory variability in the diameter of subclavian vein.

In both responders and non-responders groups, volume expansion significantly increased SVI, while significantly decreased IJV-CI and SCV-CI (p < 0.05) (Table 2) (Figure 2). Before the fluid challenge, IJV-CI and SCV-CI were significantly higher in responders than in non-responders (p < 0.05) (Table 2). After fluid challenge, SCV-CI and SVI were still significantly higher in responders than in non-responders (p < 0.05) (Table 2). Both MAP and HR were not significantly different between the two groups before and after the fluid challenge (Table 2).

**Table 2:** Hemodynamic variables before and after fluid challenge.

	Responders group (n = 43)		Non-responders group (n = 37)		P value	P value
	Before	After	Before	After	Before	After
IJV-CI (%)	31.7 ± 8.4	18.6 ± 6.2∗	19.1 ± 7.1#	15.8 ± 9.2∗	0.0002	0.119
SCV-CI (%)	30.7 ± 11.7	21.0 ± 7.4∗	20.5 ± 9.5#	15.2 ± 7.2∗#	0.0006	0.001
SVI (ml m^−2^)	36.5 ± 7.3	46.2 ± 8.1∗	39.0 ± 8.3	41.2 ± 8.5#	0.152	0.009
MAP (mmHg)	84.3 ± 13.2	80.9 ± 13.3	82.5 ± 12.6	82.1 ± 11.4	0.501	0.445
HR (beat min^−1^)	85.4 ± 11.1	84.3 ± 11.2	83.6 ± 11.6	82.2 ± 12.8	0.538	0.669
CO (l min^−1^)	5.1 ± 1.2	6.8 ± 1.5	5.4 ± 1.5	5.7 ± 1.3	0.0001	0.013
The spread of spinal anesthesia	5.0 ± 0.8	4.8 ± 0.7	4.8 ± 0.7	4.6 ± 0.6	0.001	0.003

Data are reported as mean ± SD.

IJV-CI: the respiratory variability in the diameter of internal jugular vein; SCV-CI: the respiratory variability in the diameter of subclavian vein; SVI: stroke volume index; HR: heart rate; MAP: mean arterial pressure; CO: cardiac output.

∗p < 0.05 compared with before fluid challenge. #p < 0.05 compared with Responders group.

**Figure 3:**
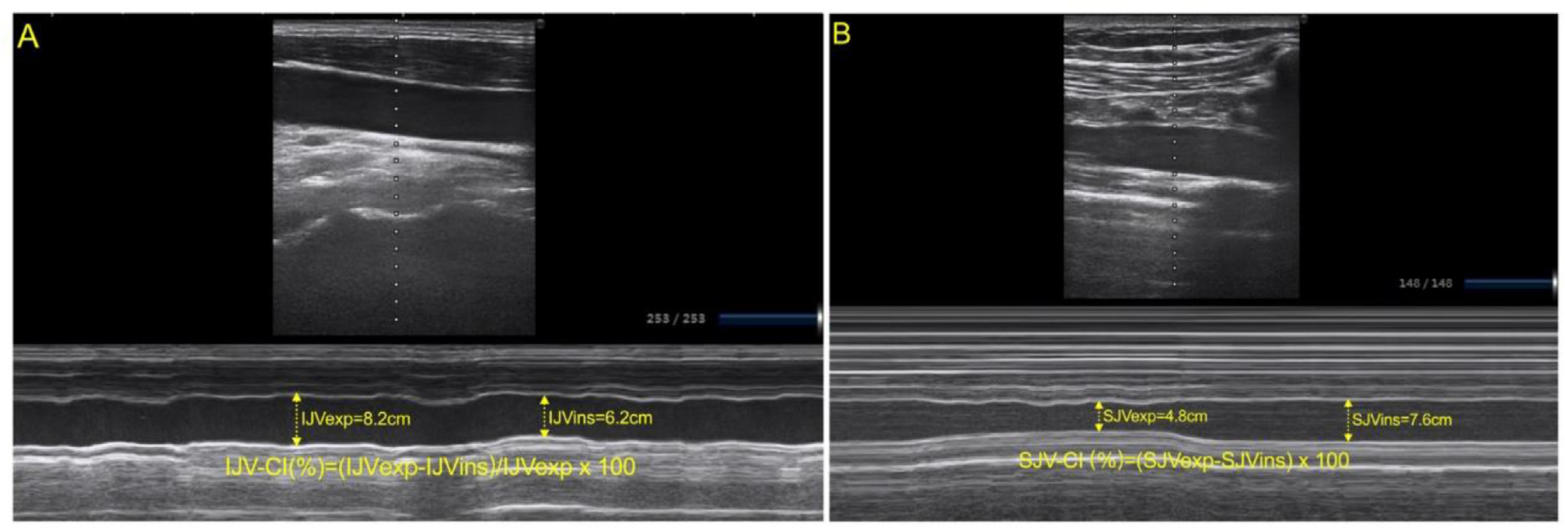
Example ultrasound images of IJV-CI, IJV-SV, and SCV-CI. IJV-CI (A) was measured at the level of the cricoid cartilage and calculated by IJV-CI (%) = (IJVexp-IJVins)/IJVexp × 100 [8]. SCV-CI (B) were measured perpendicular to the clavicle long axis and calculated by SCV-CI(%) = (SCVexp-SCVins)/SCVexp× 100 [9]. IJV-CI: the respiratory variability in the diameter of internal jugular vein; SCV-CI: the respiratory variability in the diameter of subclavian vein.

### 3.3 The ability of IJV-CI and SCV-CI to predict fluid responsiveness

IJV-CI and SCV-CI proved to be the independent predictors for fluid responsiveness by multivariate logistic regression, with the odds ratios of 0.843 (95% CI 0.760–0.935) and 0.870 (95% CI 0.798–0.948), respectively (Table 3). The regression equation for predicting fluid responsiveness in pregnant women is logit P = 7.586-0.201IJV-CI-0.108SCV-CI. The area under the ROC curve for IJV-CI and SCV-CI were 0.881 (95% CI, 0.808–0.953, p < 0.0005) and 0.757 (95% CI, 0.648–0.865, p < 0.0008) (Table 4). Their optimal cut-off values for IJV-CI and SCV-CI were 20.7% (sensitivity of 60%; and specificity of 79%) and 32.0% (sensitivity of 34%; and specificity of 96%), respectively (Table 4). The grey zone for IJV-CI and SCV-CI for fluid responsiveness were 20.4–32.4% and 30.4–44.6% and included 25% and 23% of the patients, respectively.

**Table 3:** Multivariate logistic regression analyses identified the factors that were independently associated with fluid responsiveness.

	B value	P value	Odds ratio (95% CI)
IJV-CI (%)	-0.171	0.001	0.843 (0.760–0.935)
SCV-CI (%)	-0.139	0.002	0.870 (0.798–0.948)

IJV-CI: the respiratory variability in the diameter of internal jugular vein; SCV-CI: the respiratory variability in the diameter of subclavian vein.

**Table 4:** Prediction of fluid responsiveness by receiver operating characteristic curves of IJV-CI, SCV-CI, and combining IJV-CI and SCV-CI.

	AUROC curve (95% CI)	P-value	Optimal cut-off value	Grey zone	Patients in grey zone (%)	Sensitivity (%) (95%)	Specificity (%) (95%)	Youden index
IJV-CI	0.881 (0.808–0.953)	<0.001	20.7%	20.4–32.4%	25 (31%)	60 (48–71)	79 (68–87)	0.637
SCV-CI	0.757 (0.648-0,865)	<0.001	32.0%	30.4–44.6%	23 (28%)	34 (24–58)	96 (89–99)	0.527
Combine IJV-CI and SCV-CI	0.907 (0.846–0.968)	<0.001	44.6%	/	/	91 (78–97)	77 (58–88)	0.664

AUROC, area under the receiver operating characteristic; CI, confidence interval; IJV-CI: the respiratory variability in the diameter of internal jugular vein; SCV-CI: the respiratory variability in the diameter of subclavian vein.

∗Optimal cut-off values were determined by maximising the Youden index.

### 3.4 The inter-observer agreement in estimating IJV-CI and SCV-CI

**Figure 4:**
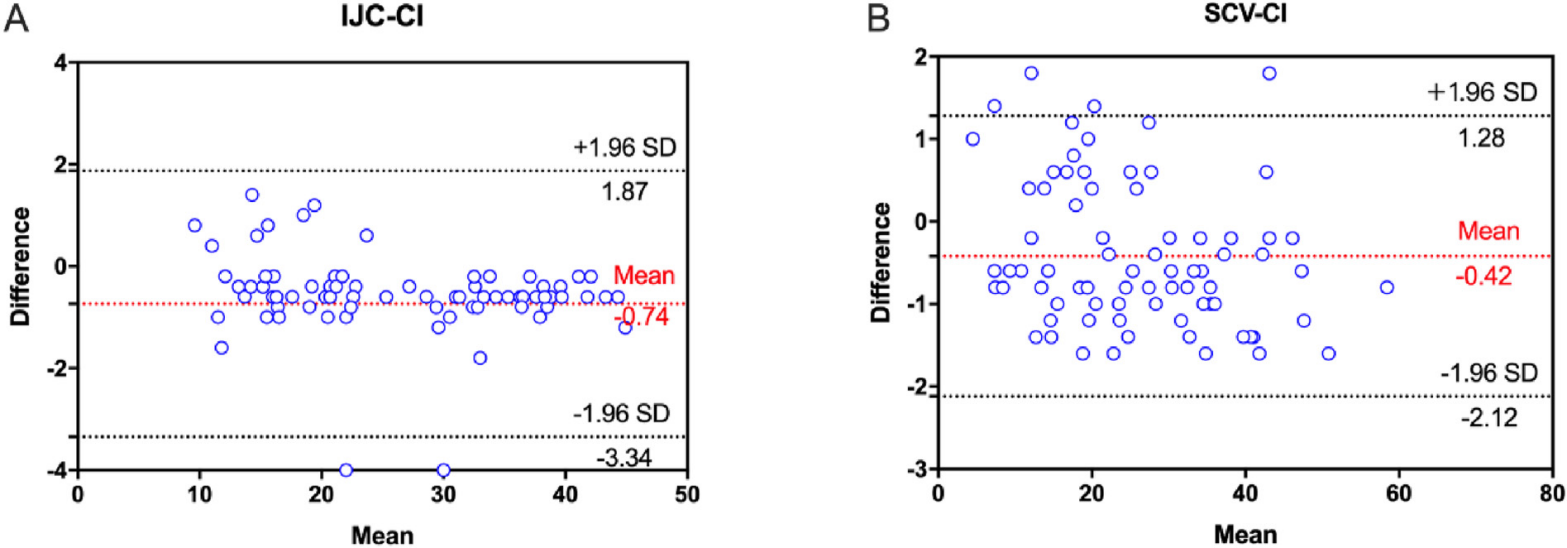
Blande-Altman plots for inter-observer agreement of IJV-CI and SCV-CI. Red dotted lines indicate the mean difference (bias), and black dotted lines indicate the 95% limits of agreement (1.96× standard deviation). IJV-CI: the respiratory variability in the diameter of internal jugular vein; SCV-CI: the respiratory variability in the diameter of subclavian vein.

For IJV-CI measurements, intra-observer variability and inter-observer variability were 4.3 (2.8) % and 3.9 (4.5) %, respectively. For SCV-CI measurements, inter-observer variability was 5.0 (2.8) % and 4.3 (3.8) %, respectively. Inter-observer reproducibility for estimating IJV-CI was excellent, with an ICC of 0.99 (95% CI, 0.981–0.998) and a CV of 38.7%. Inter-observer reproducibility for estimating SCV-CI was also excellent, with an ICC of 0.997 (95% CI, 0.996–0.998) and a CV of 45.5%. Using Bland-Altman analysis for assessing inter-observer agreement of IJV-CI and SCV-CI, the mean biases were −0.74% [with 95% limits of agreement (LOA) between −3.34 and 1.87%] and −0.42% (with 95% LOA between −2.12% and 1.28%), respectively (Figure 4).

## 4 Discussion

Fluid management is one of the most important methods to stabilize hemodynamics in parturients undergoing cesarean delivery. At present, many dynamic metrics have been used to predict volume reactivity and guide fluid therapy [17] and several noninvasive ultrasound technologies such as IVC-CI and the respiratory variability of superior vena cava diameter (SVC-CI) have been confirmed to accurately reflect fluid responsiveness in mechanically ventilated patients [3, 14, 18]. Unfortunately, the major intra-thoracic or intra-abdominal veins, such as IVC and SVC are technically difficult to visualize by transthoracic ultrasound and may not predict fluid responses during cesarean section for some reasons. Therefore, it is imperative to explore new reliable and accurate measurements as surrogate makers to predict the patient’s fluid responsiveness before volume expansion in parturients for cesarean delivery.

Known to all, changes in volume and pressure in the internal thoracic venous system can be transmitted to external thoracic veins [19] and IJV is technically easier to achieve by ultrasound. Hence, theoretically, IJV may replace IVC and SVC to predict fluid responsiveness. In previous studies, IJV-CI has been validated as a surrogate marker for IVC distensibility for predicting fluid responsiveness and guiding volume therapy in mechanically ventilated patients with sepsis or after cardiac surgery [10, 20, 21, 22, 23]. Based on a pilot study of IJV-CI in spontaneously-breathing patients with sepsis [5], our study further investigated the ability of IJV-CI to predict fluid responsiveness and found that IJV-CI was predictive of fluid responsiveness in parturients for cesarean delivery, with an AUROC curve of 0.881. The optimal cut-off value is 20.7%. Our results demonstrated that both IJV-CI was predictive of fluid responsiveness in spontaneously breathing parturients and the indices have the ability to discriminate between responders and non-responders to fluid resuscitation during cesarean delivery.

Interestingly, SVC and SCV are anatomically very close thoracic vessels and the SCV is adjacent to the pleura and the upstream of the SVC, therefore, their impact of positive pressure ventilation on pleural pressure influencing the collapsibility and swing of these large veins are very similar [24]. Importantly, it can be visualized by transthoracic ultrasound in most patients in intensive care unit (ICU) using a linear probe [24]. In general, the accessibility and ease of utilization of SCV make it possible for the assessment of intrathoracic volume status in mechanically ventilated patients [24]. In this regard, SCV-CI has proved to have a reasonable concordance with assessment using IVC-CI for both mechanical ventilation and spontaneous breathing and may be a useful adjunct to assess relative intravascular volume in patients with kidney disease [6, 25, 26]. This study is the first to further evaluate the ability of SCV-CI to predict fluid responsiveness in spontaneously breathing parturients using bedside ultrasonography for the assessment of volume status and fluid responsiveness. The result of this study is that SCV-CI proved to be the independent predictor for fluid responsiveness and SCV-CI could predict fluid responsiveness in spontaneously breathing parturients, with an AUROC curve of 0.757, which may be because that the diameter of subclavian vein is less affected by spontaneous breathing. Our data clarified that ultrasound-derived SCV-CI could be the measure of intravascular volume status and has a certain guiding role in perioperative fluid therapy in spontaneously breathing parturients.

Although we have performed a careful study, the current study still possesses some limitations. First, we should also emphasize that we conducted on a limited number of patients with potential and substantial Type 2 errors, therefore, additional studies in a larger cohort of patients are needed to identify statistically significant differences. Second, all of the parturients in our study were spontaneously breathing under epidural anesthesia without any sedation, therefore, the current results could not appreciate for mechanically ventilated patients. Next experiment should perform in parturients under general anesthesia and mechanical ventilation. Third, the parturients with pregnancy hypertension, preeclampsia, severe preeclampsia, or heart disease, which would obviously affect maternal hemodynamics and blood volume, were not enrolled in our study, we will verify the validity of these parameters in the next experiment.

In conclusion, the main conclusion of our study is that IJV-CI and SCV-CI proved to be the independent predictors for fluid responsiveness by multivariate logistic regression and ultrasound-derived IJV-CI and SCV-CI could predict fluid responsiveness in spontaneously breathing parturients, indicating that IJV-CI and SCV-CI are the reliable and accurate indicators to assess fluid responsiveness in parturients undergoing elective cesarean delivery. Combining IJV-CI and SCV-CI can be used to predict fluid responsiveness in pregnant women and the regression equation for predicting fluid responsiveness is logit P = 7.586-0.201IJV-CI-0.108SCV-CI. In the future, further efforts are needed to make rational use of ultrasound-derived IJV-CI and SCV-CI to predict fluid reactivity and guide clinical fluid therapy.

## Declarations

### Author contribution statement

Lili Xu: Conceived and designed the experiments; Wrote the paper.

Xinzhong Chen: Conceived and designed the experiments.

Jianjun Shen and Xia Tao: Performed the experiments.

Shaobing Da: Analyzed and interpreted the data.

### Funding statement

This work was supported by 10.13039/501100001809The National Natural Science Foundation of China (82271287), the Exploration Project of Zhejiang Natural Science Foundation (LY21H090006), Zhejiang Health Science and Technology Planning Project (2021KY768), and the Bureau of Chinese Medicine, Zhejiang, China (2018ZB065).

### Data availability statement

Data will be made available on request.

### Declaration of interest’s statement

The authors declare no conflict of interest.

### Additional information

No additional information is available for this paper.
